# Climate change and health: Changes in student environmental knowledge and awareness due to the implementation of a mandatory elective at the Medical Faculty of Ulm?

**DOI:** 10.3205/zma001614

**Published:** 2023-05-15

**Authors:** Laura Müller, Michael Kühl, Susanne J. Kühl

**Affiliations:** 1 University of Ulm, Medical Faculty, Institute of Biochemistry and Molecular Biology, Ulm, Germany

**Keywords:** climate change, health, education for sustainable development, enviromental knowledge, enviromental awareness

## Abstract

**Background and objectives:**

According to the World Health Organization, climate change constitutes the single greatest threat to human health. However, the health care system contributes to climate change worldwide through its high CO_2_ emissions. In order to make future physicians more aware of this issue and to expand medical education to include climate-related aspects, the mandatory 28 academic hours elective “Climate Change and Health” for students of human medicine in the preclinical study stage was implemented at the Medical Faculty of Ulm in the 2020/21 winter semester. Our accompanying study investigated

1. in what form the topic of climate change can be successfully integrated into the study of human medicine in a manner that includes student opinions and

2. whether being required to take an elective on the topic led to changes in student environmental knowledge and awareness.

**Methodology:**

Personal individual interviews were conducted with all *n*=11 students after the course in a pilot that was carried out in the 2020/21 winter semester to determine course feasibility and student acceptance. The students were also able to evaluate the course using an evaluation form and were asked to complete a questionnaire on their environmental knowledge and awareness before and after the course. The course was revised on the basis of the results and offered again in the 2021 summer semester with an intervention group (*n*=16, participation in the mandatory elective) and a comparison group (*n*=25, no participation in the mandatory elective). The intervention group was asked to evaluate the course on the evaluation form. Both groups completed the environmental questionnaire at the same time.

**Results:**

The positive feedback from students for both semesters indicates a good feasibility and acceptance of the course. Student environmental knowledge was increased in both semesters. However, there were only few observable changes in student environmental awareness.

**Conclusion:**

This paper illustrates how the topic of climate change and health can be embedded into medical studies. The students considered climate change an important topic and drew added value from the course for their future work in healthcare. The study shows that knowledge transfer at the university level is an effective way to educate the young generation on climate change and its impacts.

## Zusammenfassung

**Hintergrund und Zielsetzung:**

Laut Weltgesundheitsorganisation stellt der Klimawandel die größte gesundheitliche Bedrohung der Menschheit dar. Sogleich trägt das Gesundheitssystem über seine hohen CO_2_-Emissionen weltweit zum Klimawandel bei. Um zukünftige Medizinerinnen und Mediziner hierfür zu sensibilisieren und die medizinische Ausbildung um klimabezogene Aspekte zu erweitern, wurde an der Medizinischen Fakultät Ulm im Wintersemester (WS) 2020/21 das Wahl-Pflichtfach „Klimawandel und Gesundheit“ mit einem Umfang von 28 akademischen Stunden für Studierende der Humanmedizin im vorklinischen Studienabschnitt implementiert. Unsere begleitende Studie untersuchte,

1. in welcher Form das Thema erfolgreich in das Studium der Humanmedizin unter Einbezug der Studierendenmeinung integriert werden kann und

2. ob es durch Teilnahme an einem Wahl-Pflichtfach zum Thema zu Veränderungen in Umweltwissen und -bewusstsein bei den Studierenden kommt.

**Methodik:**

Zur Durchführbarkeit und Akzeptanz des Kurses wurden in einer Pilotrunde im WS 2020/21 mit allen *n*=11 Studierenden nach dem Kurs persönliche Einzelinterviews durchgeführt. Auch konnten die Studierenden den Kurs über einen Evaluationsfragebogen bewerten und wurden gebeten vor und nach Besuch des Kurses einen Umweltfragebogen zu Umweltwissen und -bewusstsein zu bearbeiten. Auf Basis dieser Erkenntnisse wurde der Kurs optimiert und im Sommersemester (SS) 2021 mit einer Interventions- (*n*=16, Teilnahme am Wahl-Pflichtfach) und einer Vergleichsgruppe (*n*=25, keine Teilnahme am Wahl-Pflichtfach) erneut durchgeführt. Die Interventionsgruppe bewertete den Kurs über den Evaluationsfragebogen. Beide Gruppen bearbeiteten zeitgleich den Umweltfragebogen.

**Ergebnisse:**

Die positiven Bewertungen in beiden Umläufen lassen auf eine gute Durchführbarkeit und Akzeptanz des Kurses schließen. Das Umweltwissen der Studierenden konnte in beiden Umläufen gesteigert werden. Veränderungen im Umweltbewusstsein waren hingegen nur wenig zu beobachten.

**Schlussfolgerung:**

Die vorliegende Arbeit veranschaulicht, wie das Thema Klimawandel und Gesundheit in das Medizinstudium eingebettet werden kann. Die Studierenden erachten die Thematisierung des Klimawandels als wichtig und ziehen aus dem Kurs einen Mehrwert für ihre künftige Arbeit im Gesundheitswesen. Die Studie zeigt, dass die Wissensvermittlung auf universitärer Ebene eine effektive Möglichkeit darstellt, die junge Generation zum Klimawandel und dessen Auswirkungen aufzuklären.

## 1. Introduction

### 1.1. Problem and starting point at the University of Ulm

According to the World Health Organization, climate change poses the single greatest threat to human health [1]. The following aspects are particularly important:

1. Climate change has direct and indirect effects on human physical and mental health [2]. The direct effects include the increases in injuries and deaths due to extreme weather events or the increased incidence of infectious and cardiovascular diseases [2], [3]. Indirectly, climate change also makes pathogens easier to spread [2].
2. Going forward, healthcare professionals will be confronted with the health-related consequences of climate change more frequently. At the same time, these people can become multipliers [4] and role models for climate protection.
3. The contribution of the healthcare system to climate change is significant, accounting for 4.4% of global greenhouse gas emissions. On a global average, 17% of these emissions come directly from healthcare facilities including their vehicles, 12% come from electricity, cooling and heating, and 71% come from the supply chain that includes the production, transportation and disposal of goods as well as the provision of services. If the healthcare system were a country, it would be the fifth largest greenhouse gas emitter in the world. In Germany, the healthcare system accounts for approximately 5.2% of the country’s emissions [5].

In order to educate future healthcare professionals about the effects, the steps that could be taken and possibilities for reducing greenhouse gas emissions, it is becoming increasingly important to integrate the topic of climate change and health into medical training. There is a general agreement [5], [6], [7] regarding the importance of integrating the topic of climate and health into the (medical) curriculum and even some implementation ideas [6], [8]. Several German universities offer lecture series, seminars or workshops on the topic [9], [10], [11], [12], [13], [14], [15]. The Institute for Medical and Pharmaceutical Examination Questions (IMPP) would also like to increasingly cover this topic in the state examination in the future [16]. 

To the authors' knowledge, there are currently no scientific studies available in the German-speaking region that:

1. analyze how a course on climate change and health that takes into account the opinion of students can be successfully integrated into the medical curriculum, and
2. have studied a possible change in student environmental knowledge and awareness caused by the integration of this topic into the curriculum.

The University of Ulm had only offered a few isolated courses on the topic of climate change. Aside from the work carried out by university groups and a lecture series titled “Sustainability” as an additive key qualification for all students of the University of Ulm, the topic was only marginally addressed in the medical curriculum in the form of individual events. A comprehensive and targeted event did not yet exist.

#### 1.2. Objective and questions addressed by the study

The mandatory elective "Climate Change and Health" was designed and implemented for the first time at the pre-clinical study stage in order to offer students of human medicine a comprehensive range of courses geared toward the target group. 

The present study further examined how a course on the topic of climate change geared to the target group can be integrated into the curriculum of human medicine and whether it would be accepted by the students. In addition, the study sought to find out whether participation in the mandatory elective led to changes in student environmental knowledge, which includes knowledge of the scientific basis and the current situation as well as the (health-related) consequences of climate change. Finally, the study was carried out to determine whether a participation in the compulsory elective led to changes in student environmental awareness. For a more detailed conclusion, environmental awareness was divided into three areas. The sub-area of environmental affect relates to emotional concerns, the sub-area of environmental cognition to a rational assessment, and the sub-area of environmental behavior to an active involvement in environmental issues [17].

## 2. Material and methods

### 2.1. Participants and content of the mandatory elective

Students of human medicine at the Medical Faculty of Ulm can choose a mandatory elective from several courses offered in the pre-clinical study phase (1^st^-4^th^ semester). Since the 2020/2021 winter semester, two of the authors (SJK, MK) have taught the course "Climate Change and Health" in an online format twice a year during the semester break. The course comprises 28 academic hours and is conducted as a block seminar. In terms of content, the course relates to the scientific basis as well as the causes and consequences of climate change, how climate change poses a threat to human physical and mental health, and the influence that the healthcare system has on climate change and how it can contribute to climate protection. In addition, approaches to solutions on a political, technical and individual level as well as climate-friendly alternatives for work and everyday life are discussed (see table 1 (Tab. 1)).

#### 2.2. Step-by-step implementation of the mandatory elective 

##### 2.2.1. First round (WS 2020/21)

**Figure 1:**
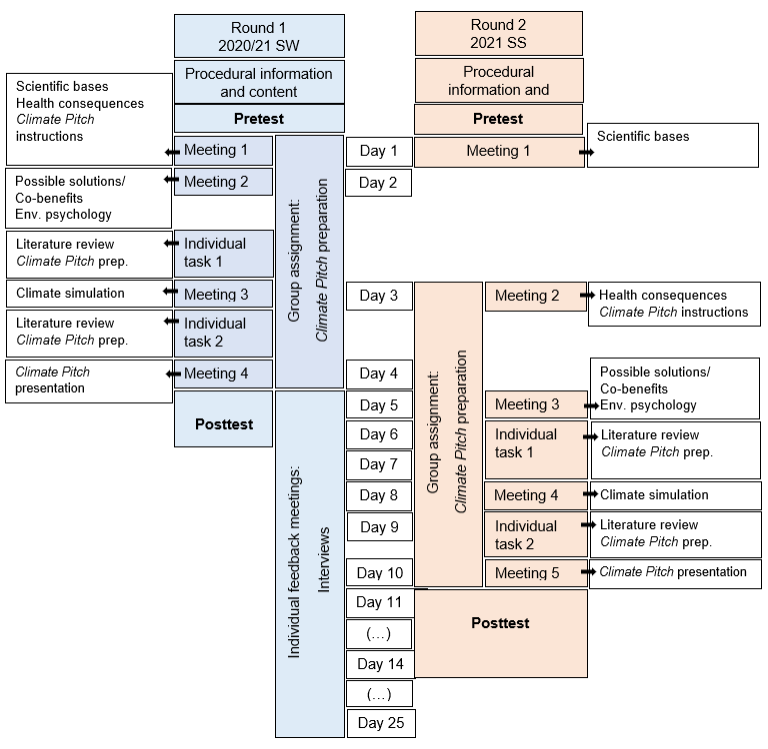
Procedure and content of the mandatory elective. The mandatory elective titled “Climate Change and Health” was scientifically accompanied by a study in the 2020/21 winter semester (WS) and the 2021 summer semester (SS). It was revised and improved on the basis of the surveys from the winter semester.

Lectures followed by discussions took place on four synchronous online dates (see figure 1 (Fig. 1)). During self-organized learning phases, teams of students used literature [18], [19], [20] to prepare an elevator pitch in which they were to convince a hospital director of a climate-neutral hospital. Interviews and questionnaires were used to scientifically monitor and evaluate the process.

##### 2.2.2. Second round (SS 2021)

Although the basic concept was retained, the course was improved upon on the basis of student feedback:

1. Organization: The time frame was extended from four to ten days to allow more time for research and group work. Five synchronous online meetings were held to allow the students to get to know each other, interact more and to offer more content.
2. Content: The content was expanded to include in-depth physiological and health aspects (influence of heat on the human body, health risks from vibrions, cercariae, blue-green algae and oak processionary moths). The term *Elevator Pitch* was changed to *Climate Pitch*.

#### 2.3. Study design and study participants

##### 2.3.1. First round (WS 2020/21)

**Figure 2:**
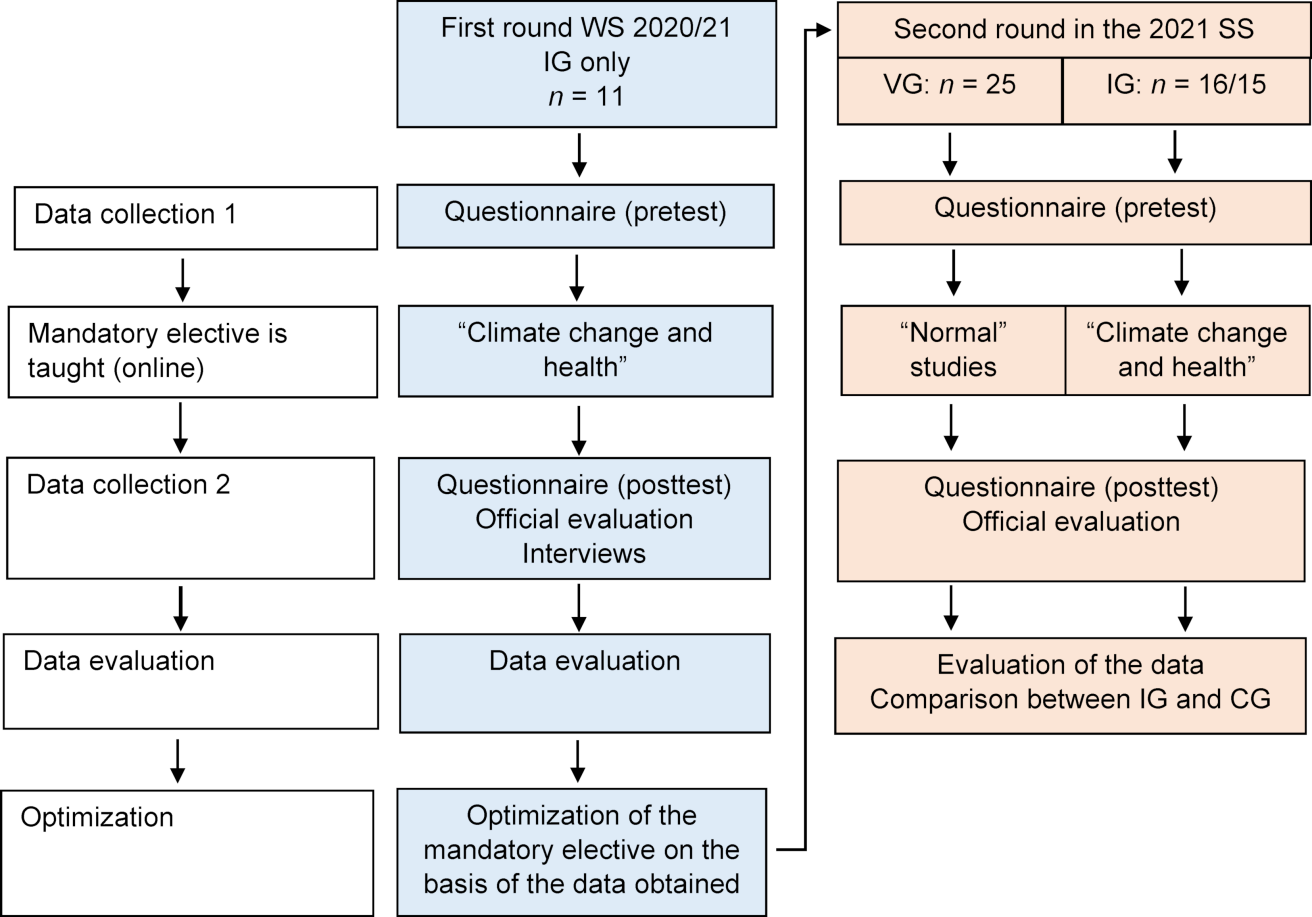
Study design. While the mandatory elective was taught in the form of a pilot study in the 2020/21 winter semester, a comparison group was also used for the surveys in the 2021 summer semester. WS=Winter Semester; SS=Summer Semester; CG=Comparison Group; IG=Intervention Group; *n*=number of students.

All participants (*n*=11) took part in the qualitative as well as the quantitative surveys (see figure 2 (Fig. 2)). Students were interviewed individually after the course. The official evaluation questionnaire of the Medical Faculty Ulm was used for an online evaluation. An environmental questionnaire on environmental knowledge and awareness was completed online before (pretest) and after the course (posttest). 

##### 2.3.2. Second round (SS 2021)

The participants of the mandatory elective evaluated the second round of the mandatory elective (Intervention Group (IG), *n*=16) via the official evaluation questionnaire as well. In addition, changes in environmental knowledge and awareness of the IG were scientifically analyzed by including a comparison group (CG) with (*n*=25). The CG consisted of students of human medicine in the preclinical study phase, who had not participated in the mandatory elective, but completed the pre- and posttests comprising the environmental questionnaire at the same time as the IG. 

#### 2.4. Data collection and analysis

##### 2.4.1. Analysis of the feasibility of the course with individual interviews

With the help of qualitative semi-structured interviews, students were surveyed for their opinions about the course taught during the winter semester 2020/21 and the integration of the topic of climate change and health into the medical curriculum. For this purpose, the authors developed a questionnaire based on the SPSS method from the publications by Helfferich [20] and Kruse [21], which serves as a guideline for qualitative surveys. The acronym SPSS stands for the key components during the development of the guide sammeln (collect), prüfen (review), sortieren (sort) and subsumieren (subsume) [22]. The questionnaire used for this study contained nine questions that addressed as many aspects as possible about the process and content of the course. Students heard the questions for the first time during the interview. The individual interviews were conducted online using the Cisco WebEx communication platform (Cisco Systems, version 41.1.2.16). They were recorded and transcribed anonymously after having obtained written and verbal consent from the interviewees. They were then coded, categorized, and evaluated using a Mayring structuring content analysis [23]. During the process, similar answers were grouped under an umbrella term in order to clearly present the numerous aspects of the questionnaires. 

##### 2.4.2. Analysis of course acceptance with an evaluation questionnaire

The official online questionnaire of the Medical Faculty Ulm was used to evaluate the course. The evaluation questionnaire designed in accordance with Rindermann [24], made it possible to evaluate the following on a Likert-type scale from 1 (strongly disagree) to 6 (strongly agree):

1. Organization, structure and format of the course,
2. Teaching motivation of the lecturers,
3. Learning objectives and content of the course,
4. Didactic implementation.

In addition, free text fields were available for praise, criticism and suggestions for improvement.

The evaluation questionnaires from both rounds were compared to assess the added value of the changes made to the mandatory elective.

##### 2.4.3. Analysis of environmental knowledge and awareness with an environmental questionnaire

**Pre- and posttest**

**Figure 3:**
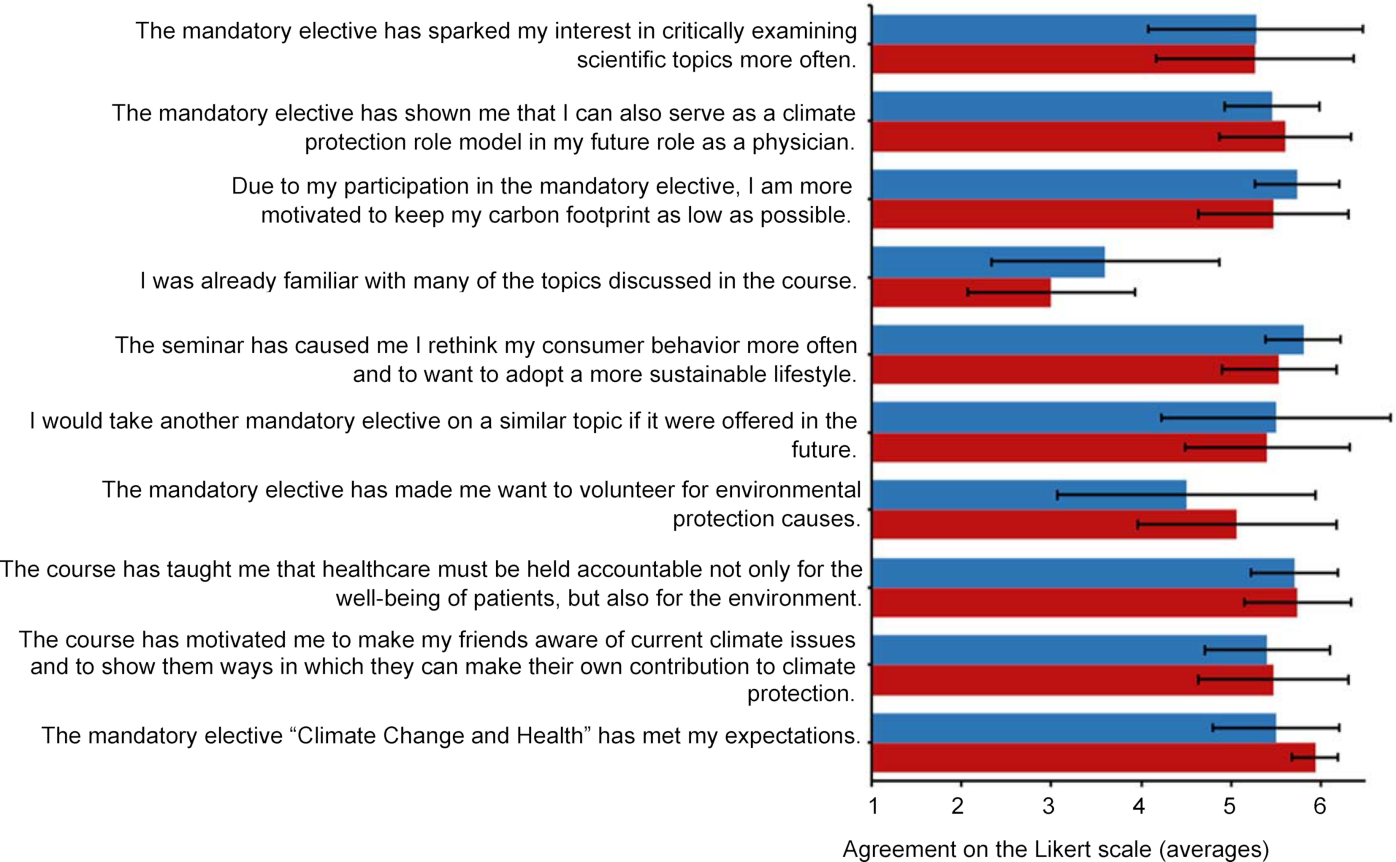
Course-specific statements in the 2020/21 winter semester (WS) (blue) and the 2021 summer semester (SS) (red). Average agreement including standard deviation of the participants (WS: *n*=11, SS: *n*=15) of the mandatory elective from 1 (strongly disagree) to 6 (strongly agree) regarding course-specific statements in the posttest.

The quantitative survey was conducted using the online tool Unipark (Tivian XI GmbH, EFS Survey, version 21.2). In the pretest, the standardized environmental questionnaire was used to collect demographic data such as age, gender, course of study, semester and voluntary involvement in environmental protection causes. The survey contained 19 specially formulated multiple-choice knowledge questions in addition to 30 questions on environmental awareness (eight questions on environmental affect, nine items each on environmental cognition and behavior (see table 2 (Tab. 2), table 3 (Tab. 3) and table 4 (Tab. 4)), and four student-specific questions using a rating on a Likert-type scale from 1 (strongly disagree) to 6 (strongly agree)). 17 of the 30 environmentally relevant questions were taken from existing questionnaires of the German Federal Environment Agency [25], the German Federal Ministry for the Environment [26] and the German Federal Agency for Civic Education [27]. In addition, the authors developed 13 new questions that were geared toward the course and its content. Using 10 additional questions that specifically addressed the content of the course, the IG was able to indicate in the posttest whether and to what extent new knowledge or additional motivation to lead a more sustainable lifestyle had been gained (see figure 3 (Fig. 3)). Students were able to express praise, criticism and suggestions in free text fields. The online survey used for the 2021 summer semester was extended by four self-developed student-specific questions that had not been included in the questionnaire used for the 2020/21 winter semester (see table 5 (Tab. 5)). 

Prior to administering the tests, 9 members of our working group checked them for unclear or misleading wording and the time it would take to complete them. All questionnaires were pseudo-anonymized and were completed voluntarily with informed consent. 

Data from the environmental questionnaires was analyzed using the IBM SPSS Statistics version 25 statistical software. For the statistical analysis of the environmental knowledge, the Wilcoxon signed-rank test was used for a significance analysis between pretest and posttest within each group (linked samples). Changes from a p-value <.05 were considered significant. 

In order to illustrate possible changes in environmental awareness, several descriptive comparisons were made: First, we examined whether there were changes between the pretests and posttests within each group. Secondly, differences between the pretests of the CG and the IG were also considered in a descriptive manner.

#### 2.5. Ethics 

The ethics committee of the University of Ulm had decided prior to the study that no approval was necessary for the scientific study.

## 3. Results

### 3.1. Results from the first round in WS 2020/21 

Since the first round was conducted as a pilot study without a comparison group, only some exemplary results are presented. While the environmental questionnaire was completed by all *n*=11 participants as part of the course, the low response rate of the evaluation questionnaire (*n*=7) is due to the fact that its completion after the course was voluntary. The analysis of the socio-demographic data from the environmental questionnaire showed that the average age of the participants was 21.8 years old. The gender distribution was equal. None of the respondents were involved in any environmental volunteer work. One participant had already attended an online seminar on a similar topic (see table 6 (Tab. 6)). 

#### 3.1.1. Results on the feasibility and acceptance of the course

To obtain more in-depth feedback about the feasibility of the course, individual interviews were conducted with all participants (*n*=11), which in summary provided the following insights: the basic concept of the course included a group work should be maintained. The course can be offered online, face-to-face, but also in a hybrid format. 82% (*n*=9) of respondents considered having attended the course valuable to their future profession. About 73% (*n*=8) of the students did not know (clearly) in advance what impact climate change would have on their work or their patients. Only 9% (*n*=1) felt sufficiently informed about the topic prior to the course (see table 7 (Tab. 7) for details).

The official evaluation questionnaire was rated very well in all four topic areas (mean values between 5.8 and 5.9; *n*=7). The comments in the environmental questionnaire (posttest) were also very positive: “Very successful introduction to an extremely relevant topic,” “I wish all people had the opportunity to be informed in such a comprehensive way and to be convinced with scientific arguments.”

##### 3.1.2. Results on environmental knowledge

A comparison of the knowledge questions showed that students significantly improved their environmental knowledge from an average of 7.09 correctly answered questions (pretest) to 12.45 (posttest) (*p*=001, *t*=-3.20).

##### 3.1.3. Results on environmental awareness

The participants were already very environmentally aware prior to the course (see table 8 (Tab. 8), A), so that only a descriptive increase in environmental awareness was observed in the posttest. On average, students agreed less with the statement “I feel powerless, because I think that average consumers can hardly contribute as much to conservation compared to industries" in the posttest than in the pretest. In terms of environmental cognition, there were significant differences in the evaluation of the statements “regular people can make a significant contribution to environmental protection with our consumption and mobility behavior” (*M_Pre_*=5.18 (*SD*=0.87), *M_Post_*=5.82 (*SD*=0.41) ) and “climate protection is indispensable for a good healthcare system” (*M_Pre_*=4.82 (*SD*=1.17), *M_Post_*=6.00 (*SD*=0.00)) (see table 8 (Tab. 8), B). The environmental behavior of the participants was already very positive in the pretest (see table 8 (Tab. 8), C). For the statement “I check several times a week for current facts on the topic of environmental and climate protection (e.g., via magazines/online/TV),” a higher level of agreement was observed on average in the posttest (see table 8 (Tab. 8), D).

Furthermore, the posttest showed a high willingness among students to implement sustainable and environmentally friendly measures in their future professional life (see figure 3 (Fig. 3)). 

#### 3.2. Results from the second round in SS 2021 

The improved course (improvements outlined in section 2.2) was taught again in the 2021 summer semester, this time with a CG. Again, the environmental questionnaire was completed by (almost) all IG participants both before (*n*=16) and after (*n*=15) the course, while only *n*=6 students evaluated the course via the voluntary evaluation questionnaire. The socio-demographic data from the environmental questionnaire revealed that the CG and the IG were comparable in terms of course of study, semester, age and gender distribution. Students in the IG were more likely to volunteer for environmental protection organizations than students in the CG (see table 9 (Tab. 9)). 

##### 3.2.1. Results on the acceptance of the course 

Since no individual interviews were conducted with the participants in the 2021 summer semester, the acceptance of the course was only determined from the evaluation questionnaire, which was rated very positively by the IG (mean values between 5.8 and 6.0; *n*=6). The statement “online teaching was able to adequately replace face-to-face teaching in terms of learning objectives” improved from 4.9 to 5.5 points (*SD*=0.80; *n*=6) compared to the evaluation from the 2020/21 winter semester.

The environmental questionnaire indicates that the course met the content expectations of the students (see figure 3 (Fig. 3)).

In addition, the participants provided positive feedback about the environmental questionnaire (posttest) in the free text fields: “This subject has its justification in every study program, because it concerns everyone,” “such and similar elective subjects should be mandatory in every study program!,” and “despite the serious (and also partly depressing) subject matter, the seminar greatly motivated me to work more on my own carbon footprint.” 

The CG also provided positive free-text comments: „Such a survey is very good,“ „it would be nice if institutions that deal with climate change and its consequences were informed about the current situation/research on a regular basis (1-2 months), possibly in the form of a lecture/meeting,” “I think it is really good that the topic is presented at the University of Ulm and that there are courses/surveys about.”

##### 3.2.2. Results on environmental knowledge 

**Figure 4:**
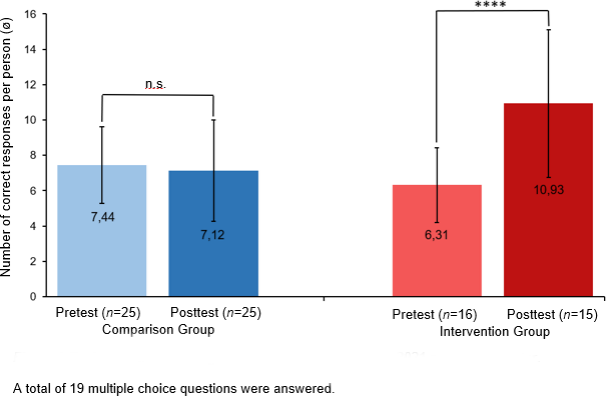
Environmental knowledge results of the students in the 2021 summer semester. A total of 19 multiple choice questions were answered. In contrast to the comparison group, a highly significant increase in knowledge is observed among students in the intervention group. ****p≤.0001; n.s.=not significant (p>.05).

The environmental knowledge analysis indicated a significant increase in the knowledge of the IG (*M_Pre_*=6.31 (*SD*=2.12), *M_Post_*=10.93 (*SD*=4.18), *p*<.0001, *t*=-7.94), while the responses of the CG to knowledge questions deteriorated descriptively (*M_Pre_*=7.44 (*SD*=2.16), *M_Post_*=7.12 (*SD*=2.86), *p*>.05, *t*=-0.53) (see figure 4 (Fig. 4)).

##### 3.2.3. Results on environmental awareness 

Clear environmental awareness differences between the CG and the IG were already evident in the pretest for the statements “it is important to me that my future employer pays attention to the environmental balance of their company/institution” (*M_Pre_* CG=4,00 (*SD*=1.08), *M_Pre_* IG=4.87 (*SD*=0.99)) and “the topic of climate change should be integrated into medical studies on a mandatory basis for all students” (*M_Pre_* CG=3.80 (*SD*=1.66), *M_Pre_* IG=4.93 (*SD*=1.10) (see table 10 (Tab. 10)).

In the area of environmental affect, the IG rated almost all statements more sustainably than the CG even in the pretest (see table 2 (Tab. 2)). 

On average, the IG agreed with the statement “It worries me when I think about the environmental conditions in which our children and grandchildren will probably have to live” more in the posttest (*M_Pre_*=5.38 (*SD*=0.72), *M_Post_*=6.00 (*SD*=0.00)) than in the pretest. Conversely, the IG agreed descriptively less with the statement “I feel powerless, because I think that average consumers can hardly contribute as much to conservation compared to industries” in the posttest than in the pretest, whereas the CG rated this statement comparably in both tests (see table 2 (Tab. 2)). No significant changes in environmental cognition were observed from the pretest to the posttest in either the IG or the CG (see table 3 (Tab. 3)). 

Regarding environmental behavior, the IG showed a high level of agreement in the pretest already, which improved even further with regard to most statements in the posttest. In the CG, a deterioration from the pretest to the posttest was observed in some cases (see table 4 (Tab. 4)). The IG agreed more strongly with the following student-specific statements in the posttest than in the pretest: “I know how I can personally contribute as much as possible to environmental and climate protection in my everyday life,” “I feel sufficiently informed about the current facts on climate change (e.g. through the media, the university)” and “the subject of climate change should be integrated as a mandatory component of medical studies for all students” (see table 5 (Tab. 5)). 

In the posttest, the IG showed high motivation to implement climate-friendly alternatives (also in their future professional life) (see figure 3 (Fig. 3)). 

## 4. Discussion

The study shows that

1. the topic of climate change and health can be successfully integrated into the preclinical human medicine curriculum as a mandatory elective subject, (with the inclusion of student opinion) in a manner that is geared to the target group and accepted by the students.
2. the environmental knowledge of students of human medicine in the pre-clinical study phase is increased by the mandatory elective.
3. the participation in the mandatory elective hardly causes any changes in the environmental awareness of students of human medicine in the pre-clinical study section.

### 4.1. Findings on the feasibility and acceptance of the course 

Compared to other courses of the Medical Faculty Ulm, the evaluation of the mandatory elective via the evaluation questionnaire was above average positive. 

The interviews from the first round provide a comprehensive picture of student wishes and expectations regarding the implementation of a course about the topic of climate change, which can also be transferred to other university locations. On the one hand, a large proportion of prospective medical students consider this course to have added value to their future profession. On the other hand, almost all of those surveyed would like to use their future career to serve as role models for climate protection. The course therefore not only serves to provide information, but also encourages the participants to take on a multiplier function. It is also interesting to note that, despite a high level of interest in the topic, the majority of students did not feel sufficiently informed about climate change before attending the course, nor did they feel able to comprehensively assess its effects. This gap must be urgently closed in order to create a basis for adequate climate-related patient care in the future. 

Overall, it is evident from all data sets that the respondents are in favor of integrating the topic of climate change into the medical curriculum. This is in line with the urging of UNESCO (2020) to integrate the topic of sustainability into all areas of education [28]. It is worth highlighting that in the posttest of the environmental questionnaire, the members of the IG were very much in favor of a mandatory integration of the topic into their studies (see table 5 (Tab. 5)). This shows that students rated the importance of the topic significantly higher after attending the course. Although the CG agreed with this statement only to a limited extent, some CG students were also in favor of integrating the topic into the curriculum. This underlines the thesis that the integration of climate topics into university teaching does not fail due to a lack of student motivation, as also described in the literature [6]. 

The comments provided on the environmental questionnaires of the IG in the 2020/21 winter semester and 2021 summer semester make it clear that the course is very instructive and important to the students. To the authors, the integration of the topic in the form of a longitudinal curriculum seems sensible. On the one hand, partial aspects of the problem could be integrated precisely into preexisting courses in order to clarify direct connections. On the other hand, it would be interesting to investigate whether constant repetition over a longer period of time leads to more significant changes in student environmental knowledge and awareness [29].

#### 4.2. Influence on the environmental knowledge of students

It was possible to increase the environmental knowledge of the students who took the mandatory elective both in the first and the second round (see figure 4 (Fig. 4)). Thus, knowledge transfer at the university level is a good way to educate the young generation about climate change and its impact. This observation is supported by the self-assessment of the students who reported feeling significantly better informed on the topic after having attended the course (see table 5 (Tab. 5)). Research has shown that increased knowledge about the impact of climate change leads to greater concern and, at the same time, increased the willingness to take action [30], [31]. Our evaluations show the same relationships: participation in the mandatory elective led to increased environmental knowledge and concern among participants, while it also strengthened their recognition of self-efficacy (see table 2 (Tab. 2) and table 5 (Tab. 5)). This suggests that basic climate change knowledge is needed before personal behavior can be adequately adapted [32].

#### 4.3. Effects on the environmental awareness of students

Student environmental affect and cognition in the IG was hardly influenced in either round. One reason could be that the participants were already very environmentally aware before taking the mandatory elective. This is supported by the voluntary registration for the course and the fact that, in the 2021 summer semester, the IG had already rated some statements in a more environmentally conscious manner than the CG had prior to the course. The question therefore arises to what extent an increase in environmental awareness is at all realistic. Nevertheless, it is worth noting that there was a strong sense of alarm among all respondents regarding the impact of climate change. This is in line with reports in the literature about a generally high level of concern in society about climate change [32]. 

Cognitive distortions can occur, since each individual also draws on personal experience in addition to facts when assessing the consequences of climate change. It is primarily information that reinforces one’s own attitude that is noticed and communicated. Oftentimes, individuals attribute more weight to their personal environmentally friendly actions (e.g., buying organic products) than to their daily environmental sins (e.g., driving a car, wasting water, producing waste). This often leads to the fact that climate change or (one’s own) climate protection efforts are subjectively assessed differently than they objectively present themselves [33]. Often, a change in beliefs only occurs when someone is personally affected by the impacts of climate change [34]. 

Our study also shows that the IG was more convinced in the posttest that they could make their own contribution to environmental protection. Nevertheless, the course had no discernible effect on the environmental behavior statements between the pretest and the posttest. This is consistent with the gap between environmental knowledge and action much described in the literature [6], [35], [36]. The IG already showed a high willingness to engage in sustainable behavior (especially regarding consumption and mobility) in the pretest. Moreover, it is uncertain whether the impulse provided by the mandatory elective taught within a short period of about two weeks is sufficient to bring about a change in behavior. Nevertheless, the higher agreement of the IG in the posttest with the statement “I know how I can personally contribute as much as possible to environmental and climate protection in everyday life” shows that the students were able to gather new ideas for establishing more sustainable behavior. In addition, a high level of motivation to implement climate-friendly measures is also evident from the course-specific questions in the posttest. 

#### 4.4. Limitations 

The changes in student environmental awareness within and between the different groups under consideration are not statistical calculations, but merely descriptive comparisons. In particular, the questionnaires of the first round were conducted with a small number of subjects and without a CG. It was, however, possible to verify the results with a CG in the second round. 

In addition, only a few students completed the official evaluation questionnaire (WS 2020/21: *n*=7, SS 2021: *n*=6), which is why these evaluations can only serve as an orientation. The authors attribute the low number of completed questionnaires to the voluntary nature of participation and the online processing of the evaluation. 

Likewise, the intervention, the implementation of the mandatory elective, took place in a rather short period of time in each case. Whether this can bring about a profound change in awareness among students is not certain. The implementation and scientific monitoring of a longitudinal curriculum could study possible long-term changes (details above) [29]. 

The voluntary enrolment of the participants in the course indicates that the IG was already aware of the importance of the topic before the intervention; this is referred to as “selection bias” in this context. Due to the fact that the students (even before taking the mandatory elective) had proactively dealt with the topic, a neutral view of the changes in environmental knowledge and awareness was not possible. 

## 5. Summary and outlook

In summary, the implementation of the mandatory elective Climate Change and Health at the Medical Faculty of Ulm was successful, as it was positively evaluated by the participants in interviews, evaluations and free text comments. Students were able to significantly increase their knowledge about the topic and gain new relevant insights for their future profession. Although only few changes in the field of environmental awareness could be observed, we recommend the implementation of similar courses at other universities. 

We have to assume, however, that it is mainly students who were already very environmentally conscious who registered for such a course. It would be interesting to investigate what results would be obtained by conducting the intervention with a (less environmentally conscious) more average student group. This would avoid the “selection bias” and make it possible to study the benefits of addressing climate change in university teaching. 

The question remains how to reach students who are not already actively engaged with climate change. For this purpose, a mandatory course on the topic as part of the medical curriculum could be considered in order to familiarize all students with the basic facts and effects of climate change. In addition, it would be interesting to investigate whether a longitudinal curriculum over a longer period of time would be more likely to bring about a change in student awareness.

## Acknowledgement

We would like to thank all those involved, especially Dr. Achim Schneider, for his support in the development of the questionnaire, and the students for completing the questionnaires.

Furthermore, we would like to thank Ute von Wietersheim (MBA, VW Translation Services) for the English translation of the manuscript.

## Competing interests

The authors declare that they have no competing interests. 

## Figures and Tables

**Table 1:**
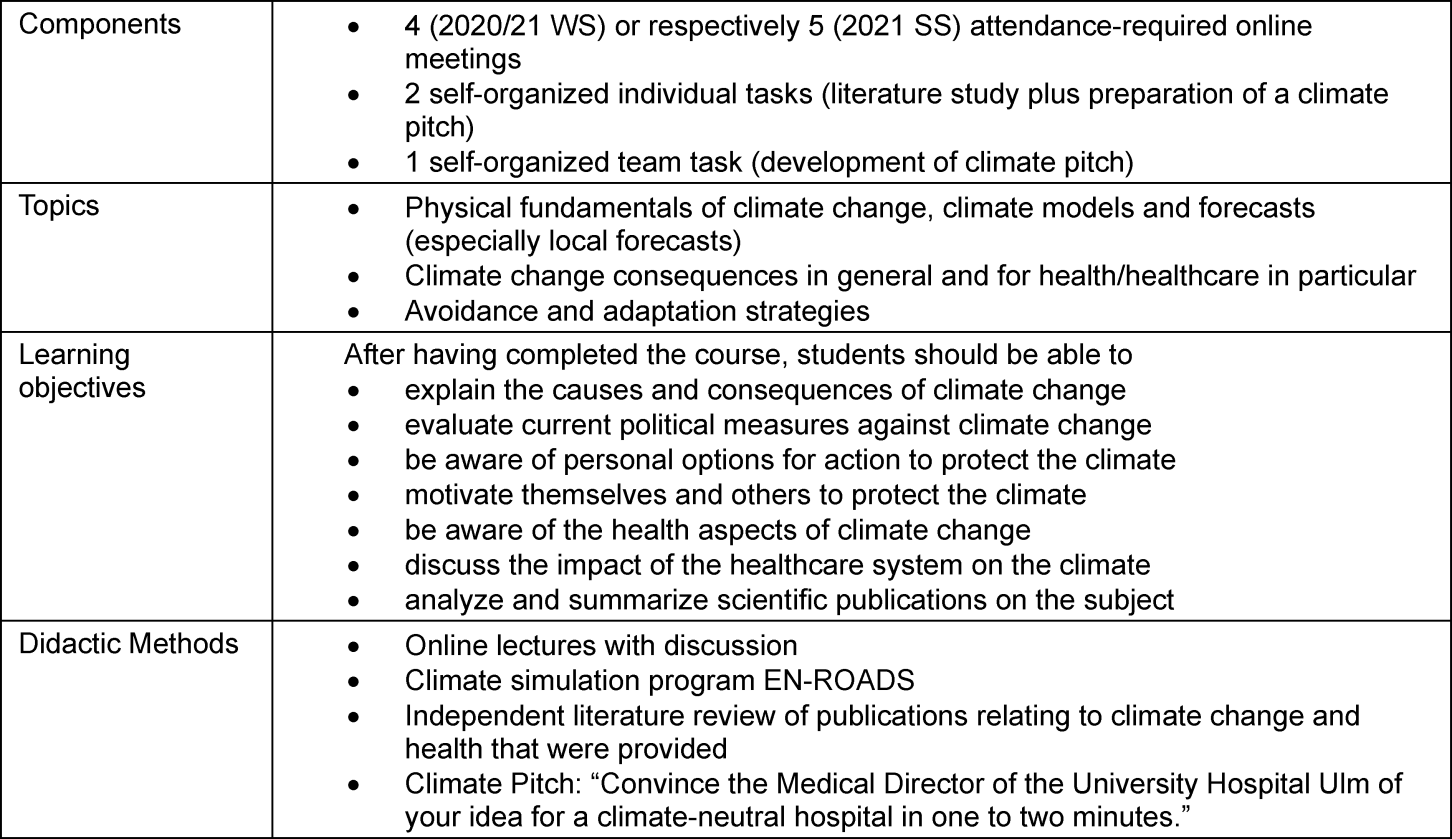
Overview of components, topics, learning objectives and methods used for the mandatory elective “Climate Change and Health”

**Table 2:**
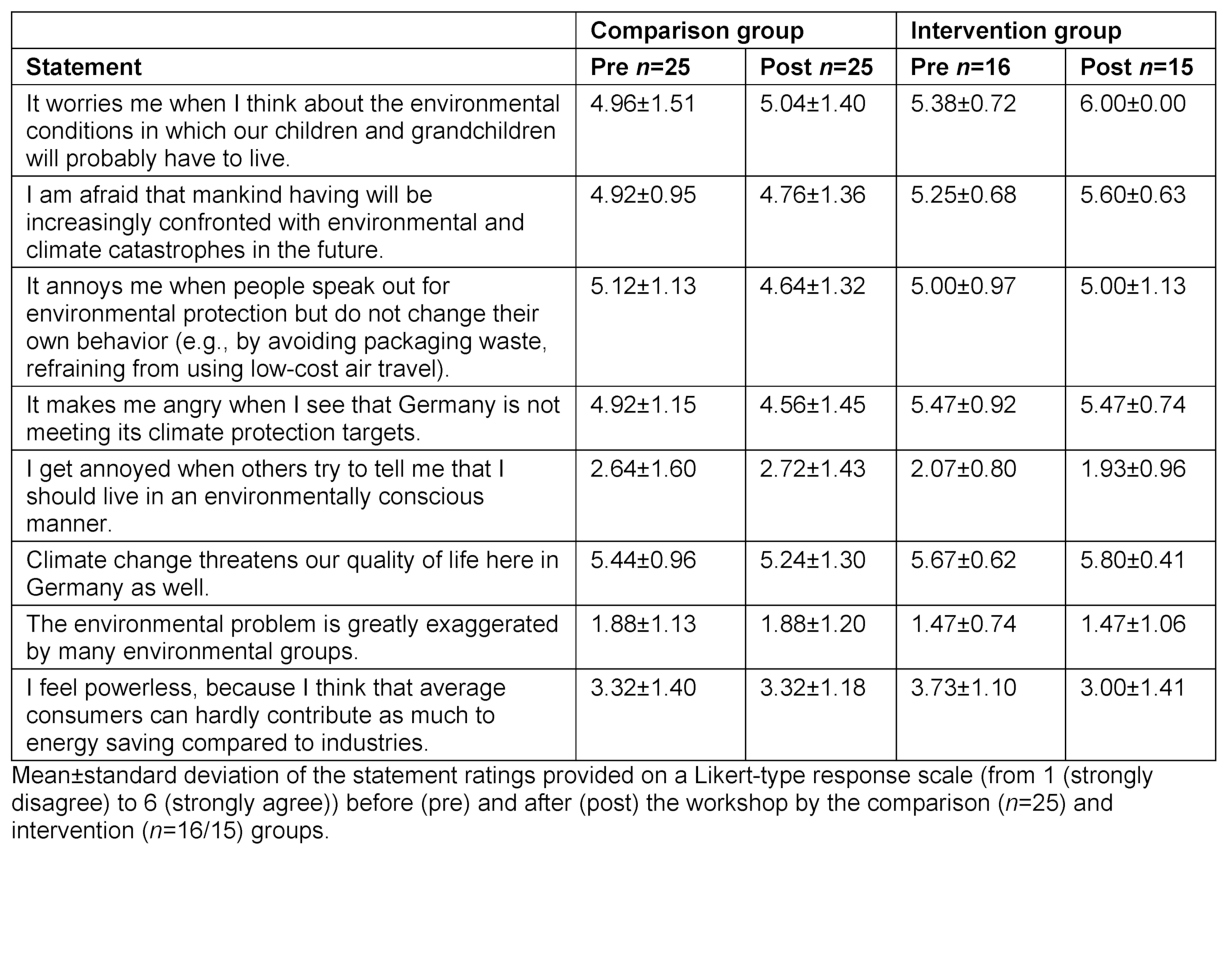
Results on environmental affect in the 2021 summer semester

**Table 3:**
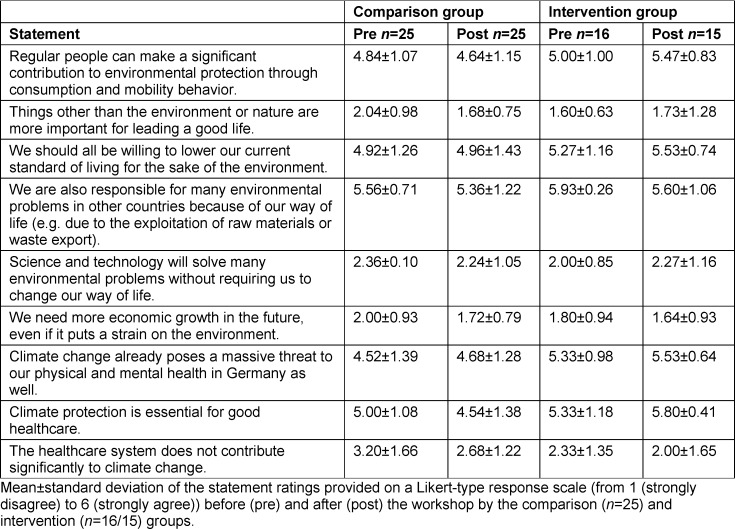
Results on environmental cognition in the 2021 summer semester

**Table 4:**
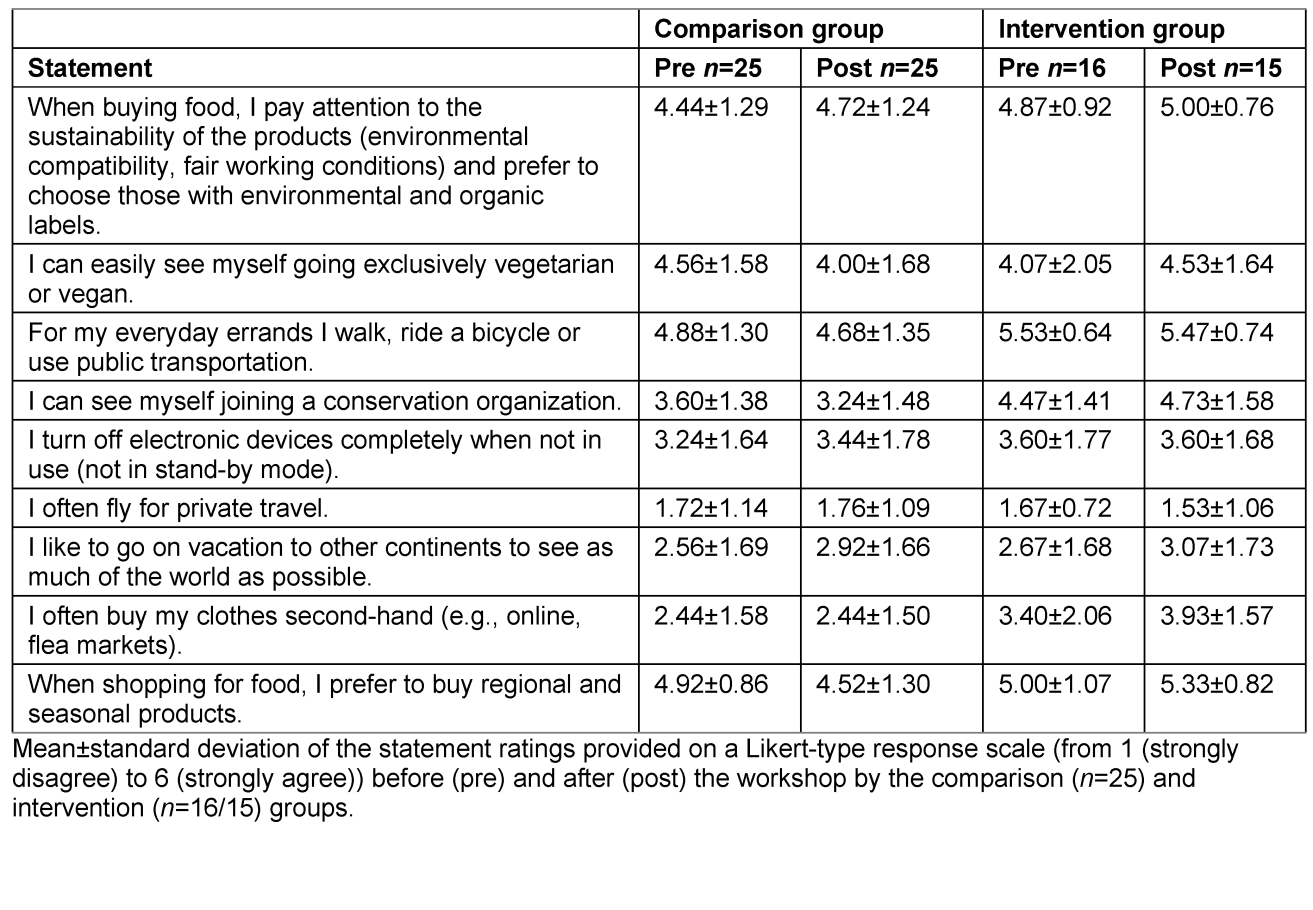
Results on environmental behavior in the 2021 summer semester

**Table 5:**
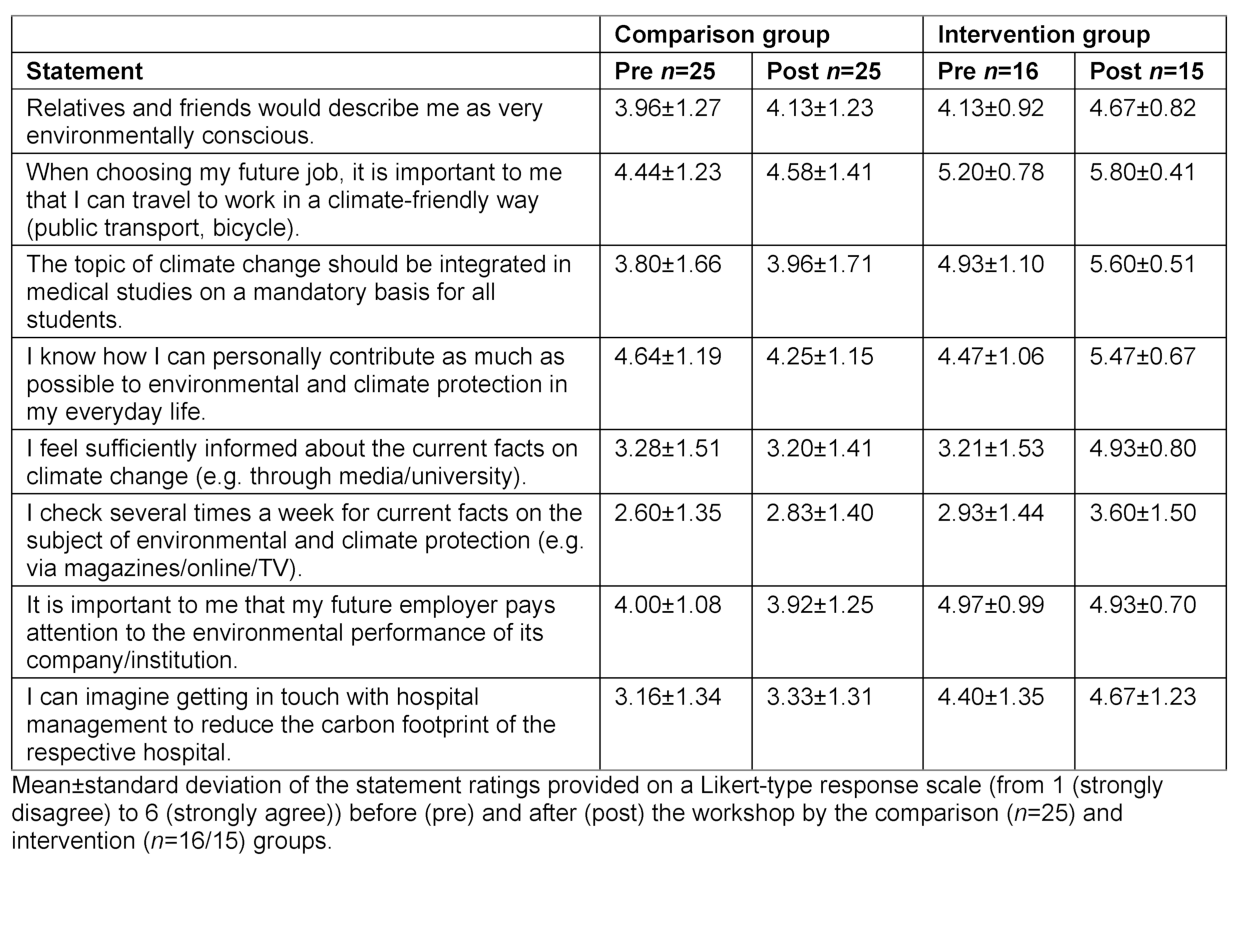
Results on student-specific statements in the 2021 summer semester

**Table 6:**
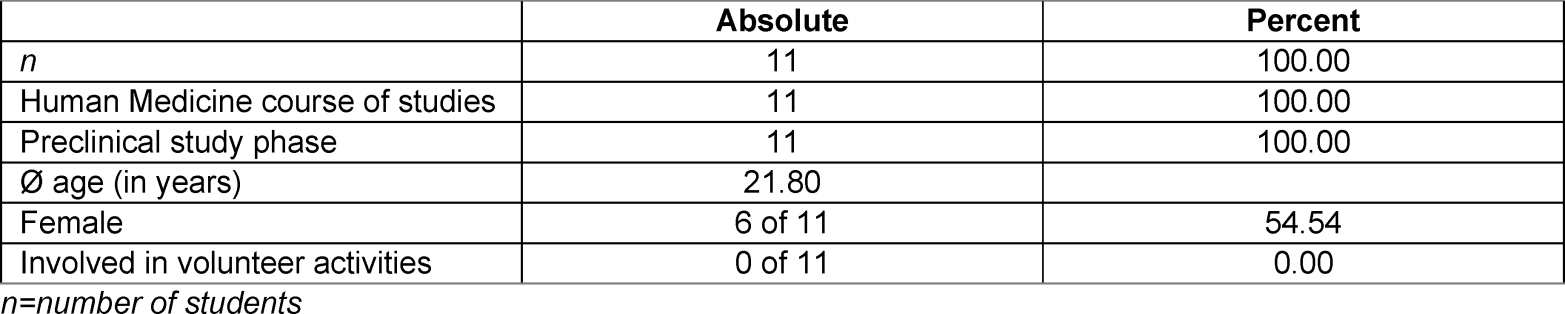
Socio-demographic data from the 2020/2021 winter semester

**Table 7:**
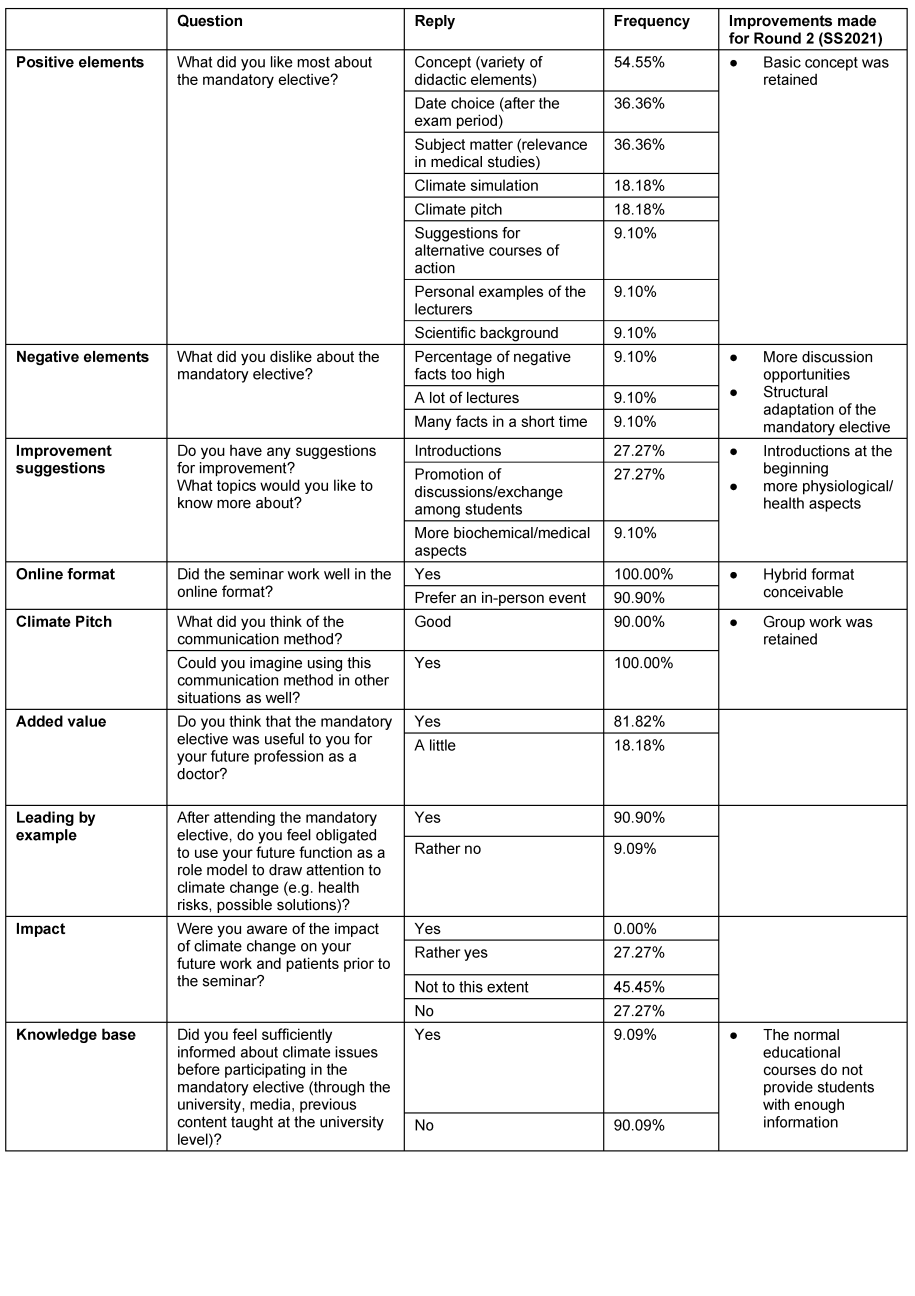
Results of the individual interviews from the 2020/2021 winter semester. All participants (*n*=11) were interviewed in person at the end of the mandatory elective. The students heard the questions for the first time during the interview and had to answer spontaneously. The findings were used to improve upon the mandatory elective.

**Table 8:**
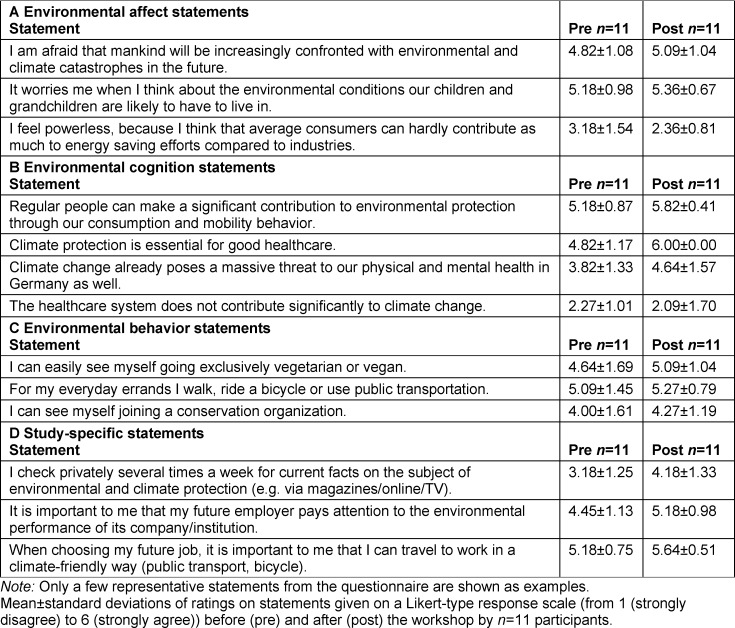
Results on environmental cognition in the 2020/21 winter semester

**Table 9:**
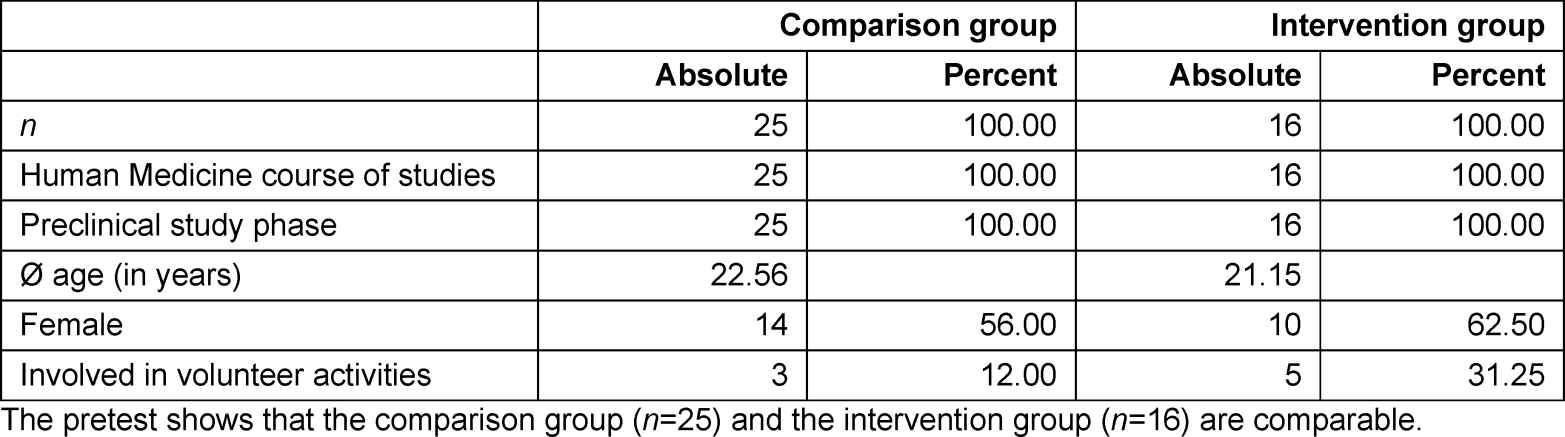
Socio-demographic data from the 2021 summer semester

**Table 10:**
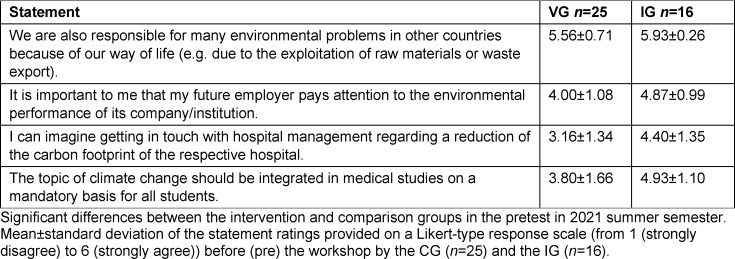
Differences in environmental awareness between the comparison and intervention groups in the pretest

## References

[1] World Health Organization (2021). Climate change and health.

[2] Bunz M, Mücke HG (2017). Klimawandel – physische und psychische Folgen. Bundesgesundheitsblatt Gesundheitsforschung Gesundheitsschutz.

[3] Eis D, Helm D, Laußmann D, Stark K (2010). Klimawandel und Gesundheit - ein Sachstandsbericht.

[4] Mücke HG, Straff W, Faber M, Haftenberger M, Laußmann D, Scheidt-Nave C, Stark K (2013). Klimawandel und Gesundheit. Allgemeiner Rahmen zu Handlungsempfehlungen für Behörden und weitere Akteure in Deutschland.

[5] Health Care Without Harm (2019). Health Care’s Climate Footprint.

[6] Filho WL, Sima M, Sharifi A, Luetz JM, Lange Salvia A, Mifsud M, Olooto FM, Djekic I, Anholon R, Rampasso I, Donkor FK, Dinis MA, Klavins M, Finnveden G, Chari MM, Molthan-Hill P, Mifsud A, Sen SK, Lokupitiya E (2021). Handling climate change education at universities: an overview. Environ Sci Eur.

[7] Wellbery C, Sheffield P, Timmireddy K, Sarfaty M, Teherani A, Fallar R (2018). It’s Time for Medical Schools to Introduce Climate Change Into Their Curricula. Acad Med.

[8] Maxwell J, Blashki G (2016). Teaching About Climate Change in Medical Education: An Opportunity. J Public Health Res.

[9] Leibniz Universität Hannover (2020). Herausforderung Klimawandel.

[10] Universität Bonn (2021). Aspekte der Erderwärmung – Ringvorlesung zum Klimawandel an der Universität Bonn.

[11] Universität zu Köln (2021). Ringvorlesung: Klima im Wandel.

[12] Charité – Universitätsmedizin Berlin (2019). Erste Professur für Klimawandel und Gesundheit.

[13] Universität Augsburg (2021). Forschung.

[14] Universität Würzburg, Lernklinik (2021). Seminarreihe Planetare Gesundheit: Klima. Umwelt. Gesundheit.

[15] Universität Frankfurt (2021). Klimawandel und Gesundheit.

[16] Institut für medizinische und pharmazeutische Prüfungsfragen (2021). Klimafolgen und Diversity in den medizinischen Staatsexamina verankern.

[17] Geiger S, Holzhauer B (2020). Weiterentwicklung einer Skala zur Messung von zentralen Kenngrößen des Umweltbewussteins.

[18] Nikendei C, Bugaj TJ, Nikendei F, Kühl SJ, Kühl M (2020). Klimawandel: Ursachen, Folgen, Lösungsansätze und Implikationen für das Gesundheitswesen. Z Evid Fortbild Qual Gesundhwes.

[19] Climate Service Center Germany (GERICS) (2020). Gesundheit und Klimawandel. Handeln, um Chancen zu nutzen und Riskien zu minimieren.

[20] Helfferich C (2009). Die Qualität qualitativer Daten: Manual für die Durchführung qualitativer Interviews.

[21] Kruse J (2015). Qualitative Interviewforschung. Ein integrativer Ansatz.

[22] Zentrum für LehrerInnenbildung (2005). Die SPSS-Methode der Leitfadenerstellung nach Cornelia Helfferich.

[23] Mayring P, Mey G, Mruck K (2020). Qualitative Inhaltsanalyse. Handbuch qualitative Forschung in der Psychologie.

[24] Rindermann H (2001). Lehrevaluation. Einführung und Überblick zu Forschung und Praxis der Lehrveranstaltungsevaluation an Hochschulen; mit einem Beitrag zur Evaluation computerbasierten Unterrichts.

[25] Scholl G, Gossen M, Holzhauer B, Schipperges M (2016). Mit welchen Kenngrößen kann Umweltbewusstsein heute erfasst werden?.

[26] Williams H, Benthin R, Gellrich A (2019). Umweltbewusstsein in Deutschland 2018.

[27] Bundeszentrale für politische Bildung (bpb) (2007). M 03.04 Musterfragebogen „Umweltbewusstsein und Klimaschutz in ….“.

[28] Deutsche UNESCO-Kommision (2020). UNESCO-Programm „BNE 2030“.

[29] Molthan-Hill P, Worsfold N, Nagy GJ, Leal Filho W, Mifsud M (2019). Climate change education for universities: A conceptual framework from an international study. J Clean Prod.

[30] Milfont TL (2012). The interplay between knowledge, perceived efficacy, and concern about global warming and climate change: a one-year longitudinal study. Risk Anal.

[31] Europäische Union (2008). Attitudes of European citizens towards the environment.

[32] Europäische Union (2017). Attitudes of European citizens towards the environment.

[33] Nikendei C (2020). Klima, Psyche und Psychotherapie. Psychotherapeut.

[34] Sloggy MR, Suter JF, Rad MR, Manning DT, Goemans C (2021). Changing climate, changing minds? The effects of natural disasters on public perceptions of climate change. Clim Change.

[35] Kollmuss A, Agyeman J (2002). Mind the Gap: Why do people act environmentally and what are the barriers to pro-environmental behavior?. Environ Educ Res.

[36] Palomo-Vélez G, van Vugt M (2021). The evolutionary psychology of climate change behaviors: Insights and applications. Curr Opin Psychol.

[37] Watts N, Amann M, Arnell N, Ayeb-Karlsson S, Beagley J, Belesova K, Boykoff M, Byass P, Cai W, Campbell-Lendrum D, Capstick S, Chambers J, Coleman S, Dalin C, Daly M, Dasandi N, Dasgupta S, Davies M, Di Napoli C, Dominguez-Salas P, Drummond P, Dubrow R, Ebi KL, Eckelman M, Ekins P, Escobar LE, Georgeson L, Golder S, Grace D, Graham H, Haggar P, Hamilton I, Hartinger S, Hess J, Hsu SC, Hughes N, Jankin Mikhaylov S, Jimenez MP, Kelman I, Kennard H, Kiesewetter G, Kinney PL, Kjellstrom T, Kniveton D, Lampard P, Lemke B, Liu Y, Liu Z, Lott M, Lowe R, Martinez-Urtaza J, Maslin M, McAllister L, McGushin A, McMichael C, Milner J, Moradi-Lakeh M, Morrissey K, Munzert S, Murray KA, Neville T, Nilsson M, Sewe MO, Oreszczyn T, Otto M, Owfi F, Pearman O, Pencheon D, Quinn R, Rabbaniha M, Robinson E, Rocklöv J, Romanello M, Semenza JC, Sherman J, Shi L, Springmann M, Tabatabaei M, Taylor J, Triñanes J, Shumake-Guillemot J, Vu B, Wilkinson P, Winning M, Gong P, Montgomery H, Costello A (2021). The 2020 report of The Lancet Countdown on health and climate change: responding to converging crises. Lancet.

